# Effectiveness of muscle strengthening exercises on the clinical outcomes of patients with knee osteoarthritis: A randomized four-arm controlled trial 

**DOI:** 10.22088/cjim.14.3.433

**Published:** 2023

**Authors:** Alireza Sadeghi, Mina Rostami, Zakiye Khanlari, Alireza Zeraatchi, Nooshin Jalili, Arezoo Karimi Moghaddam, Zhaleh Karimi Moghaddam, Ramazan Fallah, Fatemeh Sangtarash

**Affiliations:** 1 Department of Internal Medicine, School of Medicine, Vali-e-Asr Hospital, Zanjan University of Medical Sciences, Zanjan, Iran.; 2 Social Determinants of Health Research Center, Zanjan University of Medical Sciences, Zanjan, Iran.; 3 Department of Emergency Medicine, School of Medicine, Valiasr-e-Asr Hospital, Ayatollah Mousavi Hospital, Zanjan University of Medical Sciences, Zanjan, Iran.; 4 Department of Ophthalmology, School of Medicine, Vali-e-Asr Hospital, Zanjan University of Medical Sciences, Zanjan, Iran.; 5 Department of Radiation Oncology, School of Medicine, Vali-e-Asr Hospital, Zanjan University of Medical Sciences, Zanjan, Iran; 6 Department of Biostatistics and Epidemiology, School of Medicine, Zanjan University of Medical Sciences, Zanjan, Iran; 7 Rehabilation Faculty, Shahid Beheshti University of Medical Sciences, Tehran, Iran

**Keywords:** Knee osteoarthritis, Exercise therapy, Quadriceps muscle, Hamstring muscle

## Abstract

**Background:**

Knee osteoarthritis (OA) is one of the major causes of dysfunction that reduces patients’ quality of life. The aim of current study was to investigate the effectiveness of muscle strengthening exercises on the clinical outcomes of patients with knee OA.

**Methods:**

We conducted a single-blind clinical trial on 96 patients with mild to moderate knee osteoarthritis. Patients were randomly assigned to 4 groups with 24 patients and followed-up for 8 weeks. Quadriceps, hamstring and both hamstring and quadriceps strengthening exercises were received by the first (G1), second (G2) and third (G3) groups, respectively. The fourth group (G4) was the control group and did not receive any intervention. The main outcome variables were pain, physical function and morning stiffness. This study was retrospectively registered at irct.ir (Iranian Registry of Clinical Trials) with the code IRCT20220206053950N2, 2022-09-07

**Results:**

Our findings demonstrated a significant impact of interventions on VAS score, pain, and stiffness (All, p < 0.001). Nonetheless, the effect of interventions was not significant for physical function (P = 0.78). After adjusting for the potential confounders, similar results were observed. Besides, the results of Dunnett post hoc test showed that either G1 or G3 had the most decrease in VAS and WOMAC-morning stiffness scores compared to G2 and G4.

**Conclusion:**

Based on the evidence from the present study, we can recommend a combination of quadriceps and hamstring strengthening exercises as the most effective intervention to reduce pain and morning stiffness in patients with OA.

Currently, about 250 million of world population are suffering from osteoarthritis (OA). OA is a chronic musculoskeletal disorder that has long been noted as an important public health issue, especially in the elderly population as for nearly 10 to 20% of individuals > 65 years old worldwide experiencing knee OA to some extent. The prevalence and incidence of OA have also been increasing mainly due to the rapid growth of aging population and increasing prevalence of obesity, which are the major risk factors of OA (1, 2). According to epidemiological studies in Iran, the prevalence of knee osteoarthritis in the urban and rural areas is about 15.3% and 19.3%, respectively. Moreover, 24.46% of men older than 50, 57.43% of women older than 50, 57.6% of men over 70, and 79.3% of women over 70 are affected by osteoarthritis (3). Considering its high prevalence and related burdens, OA should be highly focused at all levels of healthcare system.

Knee OA results in pain, stiffness, decreased balance, and a limited physical function. Additionally, it results in several other negative consequences for the patient, such as psychological distress, tremendous socioeconomic burden, disability, and the potential need for major surgical interventions, such as joint replacement surgery (4). It has been shown that, on the whole, patients with OA represent a lower level of physical activity in comparison to the general population. Besides, lower limb muscular atrophy and weakness has often been observed among patients with knee OA, predicting the severity of pain and disability. Although, no definitive cure for OA has yet been introduced, existing evidence suggests that therapeutic exercises are one of the most advantages interventions, playing a vital role in improving post-exercise knee extension, pain, quality of life, muscular strength, range of motion (ROM), flexibility and physical function in patients with knee OA. Importantly, they are cost-effective, safe and can be prescribed even for the elderly and patients with morbid obesity (5-9). 

It is not yet completely understood what mechanisms are involved in the effectiveness of exercise therapy for knee OA. Overall, suggested theories consist of a multitude of factors involving 5 main categories of peri-articular/intra-articular-related factors, neuromuscular-related factors, general fitness and health status, and psychosocial factors (10). For example, muscle strength exercises may play a biomechanical role by decreasing the mechanical force on the articular cartilage, reducing joint loading rate, as well as improving the stability of the knee joint (11,12). Additionally, controlling lower limb movements and reducing load on the knee joint is a result of simultaneous concentric and eccentric contractions of the knee peri-articular muscles. Knee extension is a result of eccentric hamstring contraction along with concentric quadriceps contraction whereas knee flexion is a result of concentric hamstring contraction accompanying eccentric quadriceps contraction, respectively (13). It implies that performing strength exercises focusing on each of the quadriceps and hamstring muscles alone compared to exercising both muscles simultaneously may play a different part in knee joint function and stability. 

However, current guidelines recommend exercise therapy as an integral part of managing knee OA, the optimal exercise therapy or a combination of them is not yet fully identified. The majority of the studies on this subject have discovered the superiority of exercise to usual care, and therefore there are few studies to compare various types and dosage of exercises. In other words, most studies have assessed the effects of a combination of the exercises (both hamstring and quadriceps) on clinical outcomes of pain, disability and morning stiffness, while the evidence on the impacts of exercise therapy focused on each of the quadriceps and hamstring muscles alone in comparison to the mixed exercises seems inadequate (11,12). Therefore, the present study aimed at investigating the effectiveness of strengthening exercises of peri-articular muscles (hamstrings and quadriceps) on the clinical outcomes of pain, physical function, and morning stiffness among patients with knee OA. 

## Methods

**Trial design and Participants: **The present study was a single center, parallel designed four-arm (1:1:1:1) single-blind (outcome assessor and statistical analyst) randomized controlled trial performed on patients with knee OA who were referred to the Rheumatology Clinic of the Vali-E-asr Hospital, Zanjan, Iran, from March 2016 to May 2016. The present study was evaluated and approved by the Ethics Committee of the Zanjan University of Medical Sciences [ZUMS.REC.1394.301]. Written informed consent was obtained from all participants. Moreover, the control group was also instructed with the quadriceps and hamstring strengthening exercises after the end of the study. The inclusion criteria considered patients aged 40-70 years with knee osteoarthritis confirmed by a rheumatologist (grades I-III in the Kellgren-Lawrence score) who had pain, morning stiffness of shorter than 30 minutes, or knee crepitus. The exclusion criteria included concomitant involvement with musculoskeletal diseases other than knee osteoarthritis in one or both knees within the last 6 months, a history of knee joint surgery, intra-articular injection of corticosteroids or hyaluronic acid in the knee joints within the past 30 days, NSAIDS or glucosamine intake within the last 30 days, and participation in the muscle-strengthening programs for the knee joint within the last 6 months. The patients who needed medications, including NSAIDs, glucosamine, or corticosteroids, during the 8-week study period were also excluded from the study.

**Interventions: **This trial investigated the effectiveness of hamstring and quadriceps strengthening exercises on the clinical outcomes of patients with a definite diagnosis of knee OA. There were four groups in the study. The first group (G1) underwent quadriceps strengthening exercises, the second group (G2) received hamstring strengthening exercises, the third group (G3) performed strength aimed at strengthening both quadriceps and hamstring muscles groups, and the fourth group (G4) did not undergo any intervention and was considered the control group. The training for all groups was instructed by the same physiotherapist with at least 5 years of physiotherapy experience. The participants were asked to regularly perform 3 sets of exercises per day, 5 days a week for 8 weeks (3 sets/day, 10-15 repetitions). To ensure regular exercising according to the instructions, the participants were provided with written instructions of the exercise programs and a specific form to record their daily exercises during 8 weeks, in addition, they were called up once a week by the physiotherapist, encouraging patients to properly adhere to the interventions. 

**Outcomes: **Primary and secondary study outcomes, including pain, physical function, and morning stiffness, were assessed both at the baseline and 8 weeks later by an assessor blinded to each patient's group. The pain was evaluated mainly using the visual analogue scale (VAS), while the physical function and morning stiffness were assessed using the Western Ontario and McMaster (WOMAC) index. Moreover, the variables of age, gender, height and weight, were collected using a checklist.

**The Western Ontario and McMaster (WOMAC) Index: **This index is a "disease-specific" questionnaire originally used in clinical trials for assessing the clinical outcomes of osteoarthritis. Currently, it is also routinely used in clinical practice. This index is a multidimensional, self-reporting questionnaire including 3 dimensions of pain (5 items), stiffness (2 items), and physical function (17 items). The present study used its Persian version (VAS 0-100 version), in which each sub-scale scores ranging from 0-500 for pain, 0-200 for stiffness, 0-1700 for physical function, with higher scores indicating worse symptoms. The study by Naderian et al. calculated a Cronbach's alpha of 0.811 for the Persian version of this questionnaire, and a test-retest reliability with ICC value of 0.80 (14).

**Visual Analogue Scale (VAS): **This is a self-reporting scale for pain severity scoring. The assessment is performed using a 10-cm line (100 millimeter), with 0 on the left end of the line indicating "without pain" and 10 on the right side of the line as the "most severe pain." The length between the start point (left end) and the marking by the patient is measured and is interpreted as the score of pain severity (15).

**Sample Size: **The sample size needed for investigating each variable, including pain, physical function, and morning stiffness, was calculated based on the study of Jorge et al. (13), and the maximum sample size was found to be for the variable of pain. The sample size was determined using the following formula,

$n=\frac{{\left[Z_{1-\frac{\alpha }{2}}+Z_{1-\beta }\right]}^{\sigma -}}{d^{2}}$

$n_{1}=\frac{n}{1-p}$

$n_{f}=\sqrt[{2}]{{\mathrm{kn}}_{1}}$

As the variance (σ^2^) = 9, α = 0.05, β = 0.2, Sampling error (d) = 2.5 units, dropout probability (p) = 0.5 and considering a confidence interval of 95% and the statistical power of 80%, n calculated to be 12 and because we had 4 groups to compare (k = 4) thus, we found the final sample size for each group to be 24.

**Figure 1:**
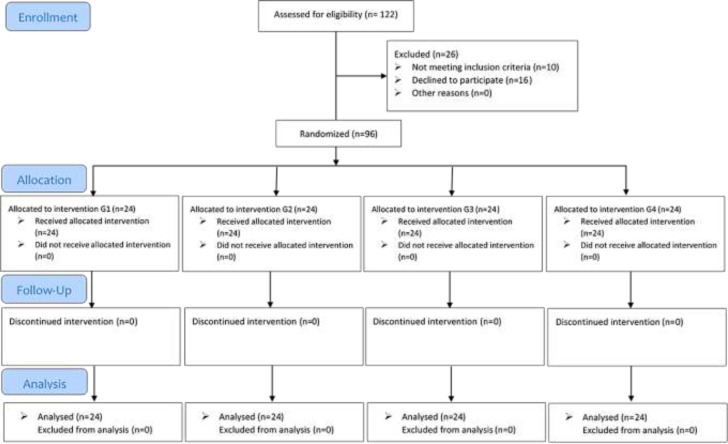
CONSORT Flow Diagram of patient’s enrolment and randomization; G1: Strength training of quadriceps muscles; G2: Strength training of hamstring muscles; G3: Strength training of both quadriceps and hamstring muscles; G4: Control

**Randomization and blinding: **After appraising subjects for eligibility criteria, a researcher who was not involved in the study used Microsoft Excel program to generate random allocation sequence through Blocked randomization with randomly varying blocks (block size 4 and 8), stratified by Kellgren-Lawrence (K & L) grade 1-2 or 3. Then, participants were randomly provided with concealed opaque envelopes identifying the assignments to each group by a research assistant with no clinical involvement in the trial. Pre and post-treatment outcome evaluations were performed by the same physiotherapist for all four groups who was blinded to the participant’s group allocation. Another physiotherapist instructed participants to do exercise therapies in the three intervention groups based on their random assignment. After the data were prepared, a statistical analyst blinded to the group allocation of the participants did the analysis. Consolidated Standards of Reporting Trial (CONSORT) diagram is shown in figure 1.

**Statistical Analysis: **Statistical analysis was conducted by IBM SPSS Statistics software [ver.26] (IBM SPSS Statistics, Armonk, NY, USA). 

The normality of the numeric variables was considered by Skewness (within ±1.5) and Kurtosis (within ±2) distribution measures. Data were presented using mean ± SD for the normal numeric variables and frequency (percent) for categorical variables. The between-group comparisons of pre and post-intervention measures and basic characteristics were done using analysis of variance (ANOVA), following a Dunnett post hoc test and chi-square tests, as applicable. For within-group comparisons, paired sample t-tests were used. The analysis of covariance (ANCOVA) was conducted in two models to assess the effect of intervention: model 1, controlling for baseline measures and model and model 2, controlling for baseline measures and confounders including age, sex, and BMI. All analyses were carried out using the per-protocol approach, and p-value less than 0.05 was considered significant. We adhered to the Consolidated Standards of Reporting Trials (CONSORT) statement.

## Results

**Basic Characteristics of the subjects: **A number of 122 patients were recruited. After evaluation for eligibility, 26 patients were excluded for not meeting the inclusion criteria and declining to participate in the study. The remaining 96(76 females, 20 males) patients were randomly allocated to the intervention arms (each n=24) and control (n=24) group. No patient was discontinued for the intervention and therefore, 96 patients were analyzed in the intervention arms (each n=24) and control (n=24) group (figure 1). There was no statistically significant difference between groups when it comes to gender, *X*2(3, N=96)= 2.52, *P*= 0.536). The mean age of the all participants was 52.70±9.15 years. The results showed no significant difference between intervention and control groups considering age (F (3, 92) = 1.78, *P*= 0.155). The average BMI of the all participants found to be 29.42±4.43 Kg/m^2^ and no significant difference was observed between groups in terms of BMI (F (3, 92) = 2.14, *P*= 0.100) (table 1).

**VAS between-group analysis: **At baseline, the mean ± SD of VAS score was 54.58±20.21, 50.21±21.79, 56.04±19.84, and 55.00±21.67 millimeter for G1, G2, G3, and G4 arms, respectively. Analysis of variance revealed no significant differences between four groups with regard to VAS score (F (3, 92) = 0.36, *P*= 0.779). However, at endpoint, the VAS score was significantly different between groups (F (3, 92) = 16.11, *p*<0.001). Besides, the results of Dunnett post hoc test showed that both G1 (MD, 95% CI, -37.54, -52.19 to -22.89,* p*<0.001) and G3 (MD, 95% CI, -36.08, -50.73 to -21.43,* p*<0.001) had the most decrease in VAS compared to G4, while G2 vs. G4 had the least reduction among intervention groups (MD, 95% CI, -21.98, -36.63 to -7.33, P=0.002). We conducted a one-way analysis of covariance, controlling for baseline measures (model 1) and baseline measures along with confounding variables (model 2). The results of model 1 showed a statistically significant difference between 4 groups (F (3, 96) = 79.53, p<0.001) with a large effect size (f^2^= 0.916) (table 2).

**VAS within-group analysis: **Paired sample t-test uncovered a statistically significant reduction in VAS mean score after 8 weeks follow-up among patients in G1 (t (23) = -7.57, *p* <0.001) with a large effect size (Cohen’s d = -1.54) and G3 (t (23) = -7.74, *p*<0.001) with a large effect size (Cohen’s d = -1.58). However, the VAS mean score decline was not significant among patients in G2 (t (23) = -1.69, *P* = 0.104) and the VAS mean score increased significantly in G4 (t (23) = 4.64, *p* <0.001) with a medium effect size (0.69) (table 3).

**WOMAC between-group analysis: **The results did not show significant differences between intervention and control groups for pain (P=0.112), stiffness (P=0.051), and physical function (P=0.579) subscales of WOMAC at baseline (table 2). Pre-intervention mean scores of WOMAC-total was 153.94±67.93, 142.14±71.92, 172.56±58.53, and 181.94±69.36 for G1, G2, G3, and G4, respectively. Considering baseline measurements of WOMAC total mean scores, there was no significant difference between groups (F (3, 92) = 1.71, *P*= 0.169). Similarly, pre-intervention mean scores of WOMAC sub-scales did not differ statistically significantly between groups (pain, P= 0.112, morning stiffness, P=0.051, physical function, P=0.579) (table 2). Mean scores of WOMAC-total at the end of the study was obtained 87.28±38.41, 95.79±47.83, 84.68±36.80, and 148.96±55.77 for G1, G2, G3, and G4, respectively which were significantly different (F (3, 92) = 10.65, *p*<0.001). Moreover, post-intervention mean scores of WOMAC pain and morning stiffness sub-scales differed statistically significantly between groups (both, *p* <0.001). Even so, groups were not significantly different in terms of WOMAC-physical function mean score (F (3, 92) =2.11, *P* = 0.103) (table 2). One-way analysis of covariance was conducted in 2 models. The results are as follows.

**WOMAC-pain: **Controlling for baseline measures (model 1) revealed that WOMAC-pain sub-scale mean score was significantly different between groups (F (3, 96) =62.95, f^2^= 0.920,* p* < 0.001). Additionally, controlling for baseline measures along with confounding variables (model 2) also showed that four groups differed statistically significantly in terms of WOMAC-pain (F (3, 92) =57.12,* p*< 0.001) with a large effect size, f^2^= 0.668. Dunnett post hoc test demonstrated that all pairwise comparisons were significant, and G1 and G3 showed the most reduction in WOMAC-pain mean score, G1 vs. G4 (MD, 95% CI, -31.56, -49.70 to -13.42, *p < 0.001*), G3 vs. G4 (MD, 95% CI, -30.20, -48.34 to -12.06,* p*<0.001) and G2 vs. G4 (MD, 95% CI, -28.43, -46.57 to -10.29, P= 0.001) (table 2).

**WOMAC-morning stiffness: **WOMAC-morning stiffness sub-scale mean score was also significantly different between groups in both model 1 (F (3, 96) =51.21, f^2^= 0.896,* p*< 0.001) and model 2 (F (3, 92) =48.39,* p*< 0.001), with a large effect size, f^2^= 0. 901. Dunnett post hoc test showed that all pairwise comparisons were significant, implying that all three types of interventions were significantly beneficial in reducing morning stiffness in comparison to the control group, however, G1 and G3 indicated the most reduction in WOMAC- morning stiffness mean score compared to the control group, G1 vs. G4 (MD, 95% CI, -28.02, -42.76 to -13.28, *p < *0.001), G3 vs. G4 (MD, 95% CI, -31.37, -46.11 to -16.63, *p* < 0.001) and G2 vs. G4 (MD, 95% CI, -21.96, -36.70 to -7.22, P = 0.002) (table 2).

**WOMAC-physical function: **Considering WOMAC-physical function, we did not find significant differences between groups in both model 1 (F (3, 96) =1.49, f^2^= 0.048,* P*= 0.219) and model 2 (F (3, 96) =1.23, f^2^= 0.042,* P*= 0.302) (table 2).

**WOMAC-total: **Finally, we performed ANCOVA model 1 and 2 for WOMAC-total and we found significant differences between groups in both model 1 (F (3, 96) =64.80, f^2^= 0.688,* p* < 0.001) and model 2 (F (3, 96) =62.29,* p*< 0.001), with a large effect size, f^2^= 0.687. Post hoc analysis showed the greatest reduction in G3 vs. G4, although all three intervention groups demonstrated significant differences compared to the control group, G1 vs. G4 (MD, 95% CI, -61.67, -92.95 to -30.40, *p < 0.001*), G3 vs. G4 (MD, 95% CI, -64.27, -95.55 to -33.00,* p*<0.001) and G2 vs. G4 (MD, 95% CI, -53.16, -84.44 to -21.89, p < 0.001) (table 2). 

**WOMAC within-group analysis: **All three intervention groups showed a significant reduction in WOMAC-pain mean score after 8 weeks follow-up (G1, t(23)=-5.33; G2, t(23)=-5.93 ;G3, t(23)=-8.17; All, p<0.001) However, for control group (G4) WOMAC-pain mean score did not significantly decline after 8 weeks (t(23)= 1.41, *P* = 0.170) (table 3). Considering WOMAC-morning stiffness, there was a significant reduction pre and post-intervention for G1 (t(23)= -4.64, *P* <0.001) and G3 (t(23)= -8.29, *p*<0.001), while both G2 (t(23)= -0.459, *P* = 0.651) and G4 (t(23)= 0.097, *P* = 0.924) mean scores did not differ significantly at the end of the study (table 3). WOMAC-physical function mean scores reduced significantly after 8 weeks of follow-up among all four groups (G1, t(23)=-4.96; G2, t(23)=-3.85 ;G3, t(23)=-5.90; G4, t(23)=-5.76; All, p <0.001) (table 3). WOMAC-total also demonstrated a significant reduction pre and post-intervention for all four groups (G1, t(23)=-8.88; G2, t(23)=-7.06 ;G3, t(23)=-12.56; G4, t(23)=-7.36; All, p<0.001) (table 3).

**Table 1:** Patients’ baseline characteristics in four groups ^a^

	**G1**	**G2**	**G3**	**G4**	P-value *
**Age, Year**	52.8 ± 9.6	49.6 ± 7.4	52.8 ± 9.7	55.7 ± 9.2	0.155
**Weight, kg**	74.50 ±11.17	79.21 ± 12.84	77 ± 9.92	82.79 ± 13.17	0.104
**BMI, kg/m2**	29.22 ± 3.77	28.54 ± 4.18	28.61 ± 4.34	31.32 ± 5.03	0.100
**Gender, Female**	20 (83.3)	18 (75.0)	21 (87.5)	17 (70.8)	0.536

G1: Strength training of quadriceps muscles; G2: Strength training of hamstring muscles; G3: Strength trainingof both quadriceps and hamstring muscles; G4: Control; BMI: Body mass index.

* Based on analysis of variance for numeric variables and chi-squared test for sex

^a ^Values are expressed as Mean ± SD or number (%)

**Table 2:** Between-group VAS and WOMAC scores pre and post-intervention

	**Pre-intervention** **mean ± SD**						**Post-intervention** **mean ± SD**							
**Outcomes**	**G1 (n=24)**	**G2 (n=24)**	**G3 (n=24)**	**G4 (n=24)**	**F** ^2^	P-value*	**G1 (n=24)**	**G2 (n=24)**	**G3 (n=24)**	**G4 (n=24)**	**F** ^2^	P-value*	P-value**	P-value***
**VAS**	54.58±20.21	50.21±21.79	56.04±19.84	55.00±21.67	0.012	0.779	29.46±21.14	45.02±22.85	30.92±19.67	67.00±21.22	0.344	**<0.001**	**<0.001**	**<0.001**
**Pain**	51.13±25.19	55.50±24.19	61.23±20.81	66.58±21.68	0.032	0.112	27.50±21.06	30.63±26.67	28.85±24.65	59.06±31.72	0.205	**<0.001**	**<0.001**	**<0.001**
**Morning stiffness**	51.67±26.57	38.54±28.42	58.54±25.77	58.23±30.88	0.080	0.051	30.56±19.37	36.63±24.43	27.22±15.88	58.59±24.57	0.254	**<0.001**	**<0.001**	**<0.001**
**Physical function**	51.15±22.55	48.10±25.47	52.79±18.78	57.13±23.58	0.021	0.579	29.22±3.77	28.54±4.18	28.61±4.34	31.30±5.05	0.034	0.103	0.219	0.302
**WOMAC-Total**	153.94±67.93	142.14±71.92	172.56±58.53	181.94±69.36	0.053	0.169	87.28±38.41	95.79±47.83	84.68±36.80	148.96±55.77	0.258	**<0.001**	**<0.001**	**<0.001**

SD: Standard deviation; MD: Mean difference; VAS: Visual Analog Scale, WOMAC: Western Ontario and McMaster Universities Arthritis Index; G1: Strength training of quadriceps muscles; G2: Strength training of hamstring muscles; G3: Strength training of both quadriceps and hamstring muscles; G4: Control group

* P-values for between-group comparisons at baseline and post-intervention were computed using one- way analysis of variance

** Model1: P-values for between-group comparisons using analysis of covariance (ANCOVA) with controlling for baseline measures

*** Model2: P-values for between-group comparisons using ANCOVA with controlling for baseline measures and confounders (including age, sex, and BMI)

**Table 3:** Within-group VAS score and WOMAC scores *

**Outcomes**	**G1 (n=24)** mean ± SD		**G2 (n=24)** mean ± SD		**G3 (n=24)** mean ± SD		**G4 (n=24)** mean ± SD	
	**Pre**	**Post**	**Pre**	**Post**	**Pre**	**Post**	**Pre**	**Post**
**VAS**	54.58±20.21	29.46±21.14	50.21±21.79	45.02±22.85	56.04±19.84	30.92±19.67	55.00±21.67	67.00±21.22
MD (95% CI)	-25.13(-31.21,-19.04)		-5.19(-11.28, 0.90)		-25.13(-31.21,-19.04)		12.00(5.91,18.09)	
d (95% CI)	-1.54(-2.23 , -0.93)		-0.34(-0.92 , 0.21)		-1.58(-2.22 , -0.92)		0.69(0.10 , 1.27)	
P-value	**<0.001**		0.104		**<0.001**		**<0.001**	
**Pain**	15.13±10.19	7.50±4.06	17.05±9.19	8.60±5.57	16.13±10.11	6.15±4.25	16.68±12.28	15.96±14.03
MD (95% CI)	-7.63(-11.56,-5.59)		-8.45(-10.05,-3.91)		-9.98(-14.12,-6.54)		-0.72(-6.46,1.47)	
d (95% CI)	-1.43(-1.61 , -0.41)		-1.21(-1.90 , -0.66)		-1.68(-2.52 , -1.17)		-0.30(-0.95 , 0.19)	
P-value	**<0.001**		**<0.001**		**<0.001**		0.170	
**Morning stiffness**	51.67±26.57	30.56±19.37	38.54±28.42	36.63±24.43	58.54±25.77	27.22±15.88	58.23±30.88	58.59±24.57
MD (95% CI)	-21.10(-29.18,-13.02)		-1.91(-9.99,6.17)		-31.32(-39.40,-23.25)		0.36(-7.72,8.44)	
d (95% CI)	-1.20(-1.44 , -0.26)		-0.09(-0.65 , 0.47)		-1.89(-2.21 , -0.92)		0.02(-0.54 , 0.58)	
P-value	**<0.001**		0.651		**<0.001**		0.924	
**Physical functioning**	51.15±22.55	29.22±3.77	48.10±25.47	28.54±4.18	52.79±18.78	28.61±4.34	57.13±23.58	31.30±5.05
MD (95% CI)	-21.93(-30.93,-12.94)		-19.56(-28.56,-10.57)		-24.18(-33.18, -15.19)		-25.83(-34.82, -16.83)	
d (95% CI)	-1.16(-1.42 , -0.24)		-0.86(-1.19 , -0.03)		-1.15(-1.42 , -0.24)		-1.40(-1.61 , -0.41)	
P-value	**<0.001**		**<0.001**		**<0.001**		**<0.001**	
**WOMAC-Total**	153.94±67.93		87.28±38.41		142.14±71.92		95.79±47.83	
MD (95% CI)	-66.66 (-82.18 , -51.13)		-46.34 (-59.23 , -33.46)		-87.88 (-102.35 , -73.42)		-32.98 (-42.25 , -23.72)	
d (95% CI)	-2.28 (-3.01 , -1.56)		-2.01 (-2.71 , -1.32)		-2.63 (-3.40 , -1.85)		-1.72 (-2.38 , -1.06)	
P-value	**<0.001**		**<0.001**		**<0.001**		**<0.001**	

SD: Standard deviation; MD: Mean difference; VAS: Visual Analog Scale, WOMAC: Western Ontario and McMaster Universities Arthritis Index; G1: Strength training of quadriceps muscles; G2: Strength training of hamstring muscles; G3: Strength training of both quadriceps and hamstring muscles; G4: Control group

* P-values for within-group comparisons were computed using paired samples t-tests

P-values for significant differences are shown in bold

**Figure 2:**
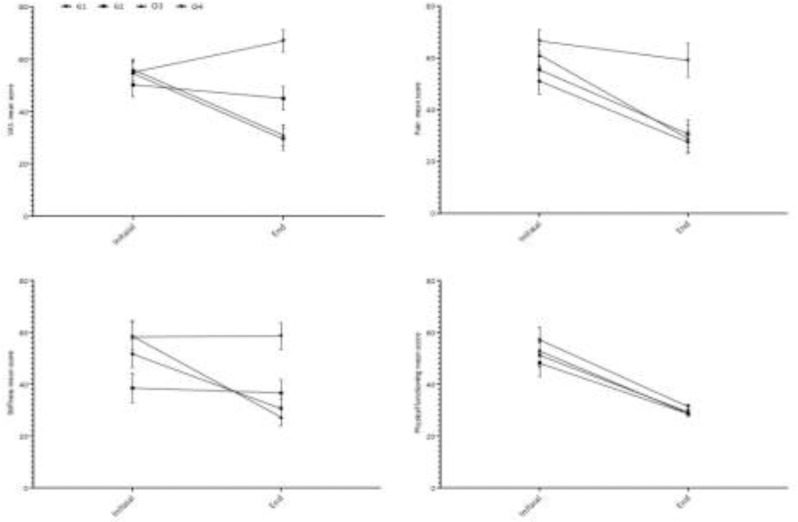
Measurements of VAS, pain, stiffness and physical functioning in study groups. G1: Strength training of quadriceps muscles; G2: Strength training of hamstring muscles; G3: Strength training of both quadriceps and hamstring muscles; G4: Control group

## Discussion

By conducting a multi-arm controlled trial we aimed to determine the effectiveness of three different types of exercise therapy on pain, physical function and morning stiffness of OA patients. We secured notable findings contributing to the ongoing debate on the optimal type of exercise therapy. In terms of VAS and WOMAC-morning stiffness, we revealed that “quadriceps” strength exercises as well as combination of “quadriceps and hamstring” strength exercises caused the greatest reduction in post-intervention mean scores compared to the control group, however, all three interventions, were significantly effective in comparison to the control group on improving pain and morning stiffness. As for WOMAC-total that all three types of exercise therapies resulted in significantly decreased mean score compared to the control group at the end of the study, with “quadriceps” as well as combination of “quadriceps and hamstring” strength exercises showing slightly higher reduction versus “Hamstring” strength exercises. Even so, we revealed that considering WOMAC-pain mean score, all three intervention arms experienced an almost equally significant decrease compared to the control group. 

Number Needed to Treat (NNT) can also be a confirmation of the present finding that for the outcome of ≥30% and ≥50% improvement in VAS score, NNT was 3 (95% CI, 1.4 , 4.8) and 4 (95% CI, 1.8 , 29.4) for the exercise therapy focused on the combination of “quadriceps and hamstring”. In contrast, for “hamstring” strength exercises, NNT ≥30% was 25, while, it was impossible to calculate the NNT for the outcome of ≥50% reduction in VAS score. Moreover, with regard to the outcome of ≥30% and ≥50% reduction in WOMAC-total for the exercise therapy focused on the combination of “quadriceps and hamstring”, NNT was calculated to be 2 (95% CI, 1.0, 1.5) and 2 (95% CI, 1.0, 1.6), respectively. Nevertheless, for “hamstring” strength exercises, for ≥30% NNT was 3 (95% CI, 1.5, 5.3) and for ≥50% was 25. Patients’ physical function in all four groups significantly improved. Similarly, WOMAC-total mean score significantly declined among patients in all four arms including control group, however, it actually might be affected by the significantly reduced WOMAC-physical function mean score among patients in the control group. The main culprit of the aforementioned finding might be due to some confounders that had not been considered and therefore not balanced between groups. It has been reported that in some lifestyle intervention trials in which physical activity of the control group had a significant increase, some basic and demographic characteristics of the participants such as type of job and education had not been taken into account. For instance, participants in the control group who were retired, had been shown to have a significantly improved physical activity despite not receiving any intervention (16). In a randomized controlled trial involving 30 patients, the intervention group received hip abductor strengthening exercises and the control group underwent only conventional exercises. The results showed that the physical function improved significantly in both groups, especially in the intervention group. The possible reason for this finding was reported to be the increase in normal blood flow in the vicinity of the joint due to quadriceps muscles strengthening exercises. However, control group received stretching exercises in addition to quadriceps strengthening exercises to affect tight peri-articular muscles and posterior capsule. On the other hand, considering that the improvement in physical function was significantly greater in the intervention group, which additionally received hip abductor muscle strengthening exercises, it seems that not only hamstring and quadriceps muscle strengthening exercises but also adding stretching and abductor muscle strength exercises might play a fundamental role in improving physical function in patient with knee OA (17).

Majority of controlled trials discovering this topic which has demonstrated significant superiority of exercise therapy focused on these group of muscles has followed-up the participants for more than 8 weeks, perhaps a pretty short follow-up duration could be another reason. Al-Johani et al. investigated the effect of a 12-week course of hamstring and quadriceps muscle-strength exercises on the 50-65-year-old patients with OA which was in line with ours. Strengthening quadriceps and hamstring muscles in addition to hamstring stretching exercises led to a significant improvement in terms of pain, ROM and physical function (9). A study by Jorge et al., which investigated the effectiveness of progressive resistance exercises in women with OA, reported that muscle-strengthening exercises for the knee extensor, flexor, and abductor muscles using exercise machines with free weights for 12 weeks led to a significantly reduced pain and improved physical function (18). In a study investigating the impact of appropriate exercise therapy on the physical function among the patients with OA, the intervention group received aerobic and strengthening exercises for 20 weeks, while the control group only received routine medical care. The authors reported a significantly improved physical function among patients in the intervention group compared to the control group (20). Unlike our study, which only investigated the effect of strength exercises of the peripheral muscles around the knee on OA-related clinical outcomes, aforementioned study appraised the effects of simultaneous performance of strength and aerobic exercises on these patients and showed a significant effect of exercises on physical function compared to the control group. 

Ninety-seven patients with knee OA in two groups (knee extensor strength exercise program and control) participated in the study of Hall et al. Pain and physical function of participants were measured using WOMAC index at baseline and 12 weeks later. According to the findings of the study, both outcomes improved significantly at the end of the study period. In spite of the current study, the intervention group participated in 7 physiotherapy sessions in addition to home-based exercises. Moreover, the study period was 12 weeks, which is longer than the follow-up duration we considered. (20). The main limitation of the present study was a relatively short follow-up period which may affect the results of the study, so,in a longer follow-up period, the effect of these exercises on the physical function improvement might have been significant. Therefore, it is suggested to perform studies with longer study duration to evaluate the long-term effects of such exercises. In summary, the concomitant performance of hamstring and quadricep muscle strengthening exercises by the patients with knee osteoarthritis was the most effective intervention, leading to reduced pain, morning stiffness and WOMAC-total mean score compared to the control group.

## References

[1] Chow YY, Chin KY (2020). The Role of inflammation in the pathogenesis of osteoarthritis. Mediators Inflamm.

[2] Liu Y, Ding W, Wang HL (2019). Gut microbiota and obesity-associated osteoarthritis. Osteoarthritis Cartilage.

[3] Wellsandt E, Golightly Y (2018). Exercise in the management of knee and hip osteoarthritis. Curr Opin Rheumatol.

[4] Yilmaz M, Sahin M, Algun ZC (2019). Comparison of effectiveness of the home exercise program and the home exercise program taught by physiotherapist in knee osteoarthritis. J Back Musculoskelet Rehabil.

[5] Haq SA, Davatchi F (2011). Osteoarthritis of the knees in the COPCORD world. Int J Rheum Dis.

[6] Alkhawajah HA, Alshami AM (2019). The effect of mobilization with movement on pain and function in patients with knee osteoarthritis: a randomized double-blind controlled trial. BMC Musculoskelet Disord.

[7] Lawford BJ, Bennell KL, Allison K, Schwartz S, Hinman RS (2022). Challenges with strengthening exercises for people with knee osteoarthritis and comorbid obesity: a qualitative study with patients and physiotherapists. Arthritis Care Res (Hoboken).

[8] Amin S, Baker K, Niu J (2009). Quadriceps strength and the risk of cartilage loss and symptom progression in knee osteoarthritis. Arthritis Rheum.

[9] Al-Johani AH, Kachanathu SJ, Ramadan Hafez A (2014). Comparative study of hamstring and quadriceps strengthening treatments in the management of knee osteoarthritis. J Phys Ther Sci.

[10] Beckwée D, Vaes P, Cnudde M, Swinnen E, Bautmans I (2013). Osteoarthritis of the knee: why does exercise work? A qualitative study of the literature. Ageing Res Rev.

[11] Bartholdy C, Juhl C, Christensen R (2017). The role of muscle strengthening in exercise therapy for knee osteoarthritis: A systematic review and meta-regression analysis of randomized trials. Semin Arthritis Rheum.

[12] Fransen M, McConnell S (2008). Exercise for osteoarthritis of the knee. Cochrane Database Syst Rev.

[13] Aslan Ö, Batur EB, Meray J (2020). The Importance of functional hamstring/quadriceps ratios in knee osteoarthritis. J Sport Rehabil.

[14] Nadrian H, Moghimi N, Nadrian E (2012). Validity and reliability of the Persian versions of WOMAC Osteoarthritis Index and Lequesne Algofunctional Index. Clin Rheumatol.

[15] Delgado DA, Lambert BS, Boutris N (2018). Validation of digital visual analog scale pain scoring with a traditional paper-based visual analog scale in adults. J Am Acad Orthop Surg Glob Res Rev.

[16] Waters LA, Reeves MM, Fjeldsoe BS, Eakin EG (2011). Characteristics of control group participants who increased their physical activity in a cluster-randomized lifestyle intervention trial. BMC Public Health.

[17] Singh S, Pattnaik M, Mohanty P, Ganesh GS (2016). Effectiveness of hip abductor strengthening on health status, strength, endurance and six minute walk test in participants with medial compartment symptomatic knee osteoarthritis. J Back Musculoskelet Rehabil.

[18] Jorge RT, Souza MC, Chiari A (2015). Progressive resistance exercise in women with osteoarthritis of the knee: a randomized controlled trial. Clin Rehabil.

[19] de Rooij M, van der Leeden M, Cheung J (2017). Efficacy of tailored exercise therapy on physical functioning in patients with knee osteoarthritis and comorbidity: a randomized controlled trial. Arthritis Care Res (Hoboken).

[20] Hall M, Hinman RS, Wrigley TV (2018). Knee extensor strength gains mediate symptom improvement in knee osteoarthritis: secondary analysis of a randomised controlled trial. Osteoarthritis Cartilage.

